# Per- and Polyfluoroalkyl Substances (PFAS) Enhance Cholesterol Accumulation and Dysregulate Inflammatory Responses in Macrophages

**DOI:** 10.1007/s12012-025-10048-w

**Published:** 2025-07-29

**Authors:** Jack C. Connolly, Yasuhiro Ishihara, Emma Sawaya, Valerie Whitfield, Nicole Garrity, Rajveer Sohata, Mark Tsymbal, Alyssa Lundberg, Michele A. La Merrill, Jamie C. DeWitt, Allison K. Ehrlich, Christoph F. A. Vogel

**Affiliations:** 1 Center for Health and the Environment, University of California, Davis, CA 95616 USA; 2 Program of Biomedical Science, Graduate School of Integrated Sciences for Life, Hiroshima University, Hiroshima, 739-8521 Japan; 3 Department of Environmental Toxicology, University of California, Davis, CA 95616 USA; 4 Department of Environmental and Molecular Toxicology, Oregon State University, Corvallis, OR 97331 USA

**Keywords:** Cardiovascular disease, Cytokines, Foam cells, Lipids, Macrophages, PFAS, PPAR, SARS-CoV-2

## Abstract

Epidemiological studies and in vivo animal models have shown that exposure to PFAS can lead to cardiovascular toxicity and promote atherosclerosis. In this study, we explored the effects of PFOA and PFOS exposure on lipid accumulation in macrophages and analyzed critical markers of foam cell formation, which are early precursors of atherosclerotic lesions. Our results demonstrate that PFOS and PFOA enhance lipid and cholesterol accumulation in human U937-derived macrophages, which is characteristic of foam cells. PFOS and PFOA induced the activity of the peroxisome proliferator-activated receptor gamma (PPARγ) and treatment with a PPARγ antagonist partly reversed the accumulation of lipids after PFAS exposure. Furthermore, the results show that PFOS and PFOA activate (NF)-erythroid-derived 2 (E2)-related factor 2 (Nrf2) and induce markers of oxidative stress. Gene expression analysis revealed that mRNA levels of interleukin-1β (IL-1β) and plasminogen activator inhibitor-2 (PAI-2) were upregulated in a time- and concentration-dependent manner in PFOS- and PFOA-treated macrophages. The expression of other key atherosclerosis-related enzymes, including cytochrome P450 8B1 (CYP8B1) and lanosterol synthase (LSS), was downregulated, whereas the expression of cyclooxygenase 2 (COX-2) and aldo–keto reductase family 1 member C3 (AKR1C3) was induced by PFOS and PFOA. Additionally, elevated levels of matrix metalloproteinases (MMP)-1 and MMP-12 were found in PFOS- and PFOA-treated cells, which were associated with increased cell migration. Furthermore, PFOS and PFOA enhanced the expression of IL-1β when macrophages were activated; however, elevated levels of IL-6 and COX-2 in activated macrophages were repressed by PFOS and PFOA. Together, the findings indicate that PFAS exposure modifies immune responses and promotes lipid accumulation in macrophages, potentially contributing to foam cell and plaque formation in atherosclerosis.

**Graphical Abstract:**

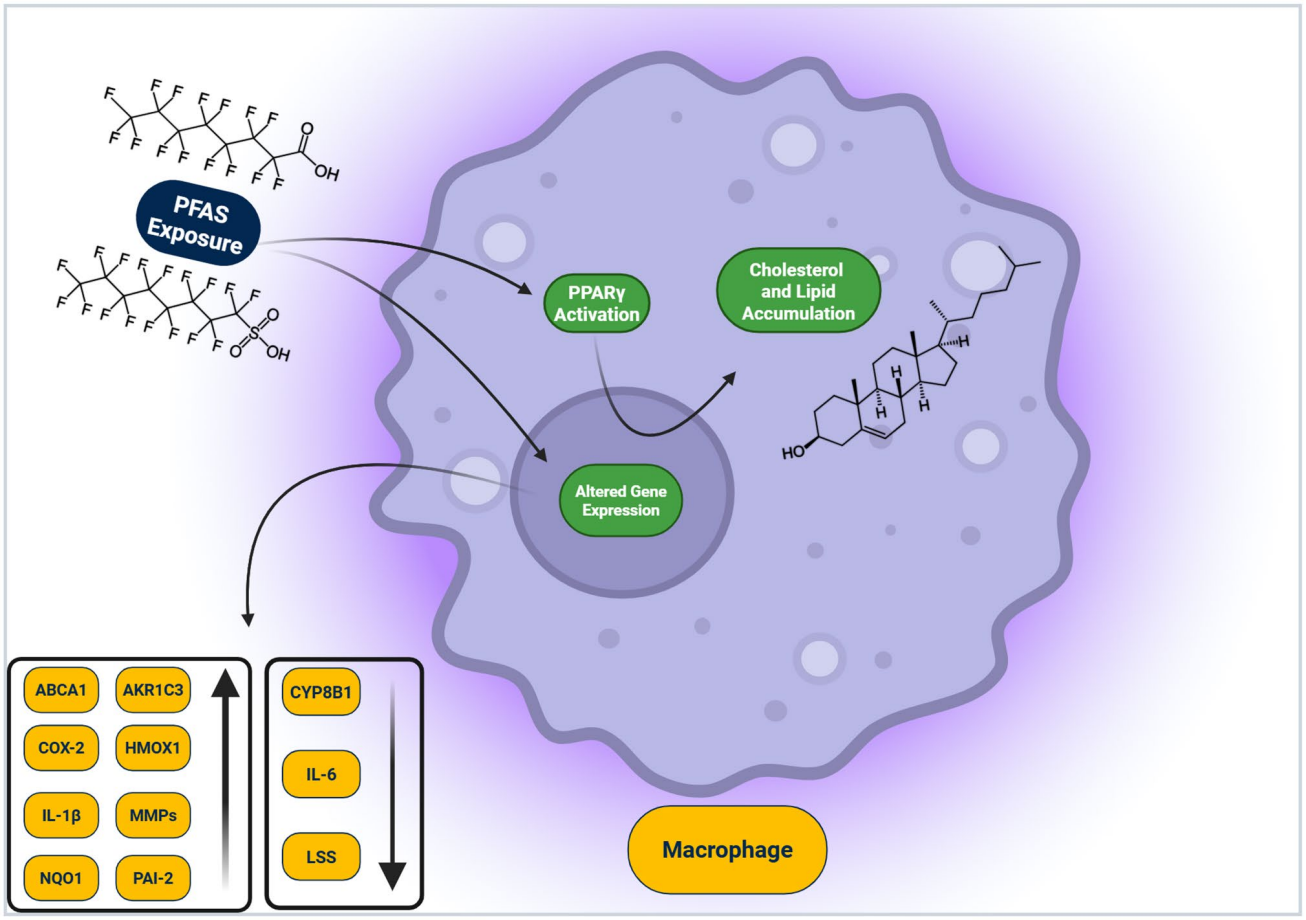

**Supplementary Information:**

The online version contains supplementary material available at 10.1007/s12012-025-10048-w.

## Introduction

Based on their stable physicochemical properties and environmental fate, many per- and polyfluoroalkyl substances (PFAS) are now widespread contaminants in the environment with a high potential for bioaccumulation in humans and wildlife. While the general human population receives a bulk of their PFAS exposure from their diets, contamination of drinking water is a significant source of exposure to PFAS chemicals for subsets of the human population [1]. Recent studies have investigated the presence of PFAS in atmospheric particulate matter (PM), identifying key sources such as industrial activities, the use of firefighting foams, and waste incineration [2–4]. The presence of PFAS in ambient PM emphasizes the inhalation exposure route, which has been underexplored compared to water and soil contamination. PFAS such as perfluorooctanesulfonic acid (PFOS) and perfluorooctanoic acid (PFOA) are amphiphilic and very stable compounds, making them desirable for use in consumer products such as surface coatings to resist heat, oil, stains, grease, and water. PFAS concentrations vary among body compartments, with the highest levels of accumulation occurring in the blood plasma, liver, and kidneys [5–8]. PFAS bind to abundant blood serum proteins including serum albumin and globulins, which affects their transport to active sites and potentially their toxicity and elimination half-lives [9].

Exposure to PFAS has been tied to a range of health problems in humans. Data from myriad published epidemiological studies report health effects such as elevated levels of cholesterol, impaired immune responses, metabolic dysfunction, and kidney cancer linked to PFAS exposure [10–14]. Numerous studies in experimental models have demonstrated dysregulation of lipid metabolism caused by PFAS exposure. A strong link between PFAS exposure and elevated cholesterol levels has been consistently observed in mechanistic studies as well as epidemiological research [15–21]. Evidence of PFAS interference with lipid metabolism stems from their interactions with cellular proteins, including several membrane proteins and liver fatty acid-binding protein (L-FABP), and nuclear receptors, such as estrogen receptor alpha (ERα), the aryl hydrocarbon receptor (AhR), and members of the peroxisome proliferator-activated receptor (PPAR) family [16]. Disturbance of lipid metabolism by PFAS exposure affects many physiological processes leading to various pathological outcomes, including cardiometabolic diseases. Changes in blood lipids are known to be high-risk factors for cardiovascular diseases (CVD) including atherosclerosis, coronary heart disease, and stroke [22]. Consequently, exposure to PFAS is associated with high levels of cholesterol, insulin dysregulation, dyslipidemia, obesity, type 2 diabetes, and CVD [23]. The underlying mechanisms leading to alterations in blood lipids through PFAS exposure in humans are not well understood. Emerging research has implicated PFAS in various aspects of CVD, particularly concerning macrophage and monocyte activation, lipid accumulation, and the progression of atherosclerosis. The altered lipid profiles found in epidemiological studies may predispose individuals to atherosclerosis by the formation of lipid-rich foam cells derived from macrophages, leading to plaque development associated with elevated levels of inflammatory cytokines [24]. Studies with bone marrow-derived macrophages and THP-1-derived macrophages reported increased levels of IL-1β mRNA and protein secretion after treatment with PFOS via activation of the AIM2 pathway [25]. A study with murine macrophages RAW264.7 cells found increased levels of inflammatory cytokines associated with apoptosis and ROS generation induced by 10 μM and 100 μM PFOA [26]. A more recent study analyzing the effects of a various PFAS molecules on THP-1 monocytes and macrophages showed that the carbon chain length and functional headgroup of a PFAS are major determinants with regard to the generation of reactive oxygen species (ROS) and cytotoxicity of PFAS in THP-1 monocytes and macrophages [27]. In this current study, we analyzed the effects of PFOS and PFOA exposure on cholesterol accumulation and the expression of atherogenic and inflammatory markers in human U937-derived macrophages.

## Materials and Methods

### Materials

Perfluorooctanesulfonic acid (PFOS) and perfluorooctanoic acid (PFOA) were kindly provided by Jamie DeWitt (Oregon State University, Corvallis, USA). The PPARγ luciferase reporter plasmid was kindly provided by Thomas Haarmann-Stemmann (Leibniz Research Institute for Environmental Medicine, Düsseldorf, Germany) and Stephen Safe (Institute of Biosciences & Technology at Texas A&M University, USA). The Nrf2 luciferase reporter construct and luciferase assay reagents were purchased from Promega (Madison, MI, USA). Dimethyl sulfoxide (DMSO) was obtained from Sigma-Aldrich (St. Louis, MO, USA). 12-O-Tetradecanoylphorbol-13-acetate (TPA) was purchased from InVivogen (San Diego, CA, USA). Recombinant SARS-CoV-2 Spike S1 protein was purchased from RayBiotech (Peachtree Corners, GA, USA). The PPARγ antagonist GW9662 and N-acetylcysteine (NAC) were purchased from Medchemexpress LLC (Princeton, NJ, USA). Other molecular biological reagents were purchased from Cayman Chemical (Ann Arbor, MI, USA) and Roche Clinical Laboratories (Indianapolis, IN, USA).

### Cell Culture and Differentiation

Human U937 monocytic cells were obtained from A.T.C.C. (Manassas, VA, USA) and maintained in RPMI 1640 medium containing 10% fetal bovine serum (GIBCO, Woodland, CA, USA) supplemented with 3.0 g/L glucose. Cell culture was maintained at a cell concentration between 2 × 10^5^ and 2 × 10^6^ cells/ml. For differentiation from monocytes into macrophages, U937 cells were treated with TPA (3 μg/mL) and allowed to adhere for 48 h in a 5% CO_2_ tissue culture incubator at 37 °C, after which they were fed with TPA-free medium. Subsequently, U937 cells were treated with PFOS or PFOA for the indicated time periods and concentrations. The range of the concentrations for PFOS and PFOA was selected on the current literature and use of PFAS in various cell culture models. In the current study, we did not observe cytotoxic effects at the highest concentration of 50 μM PFOS or PFOA.

## Cell Viability Assay

To assess the effect of PFOS and PFOA on the viability of U937 macrophages, we used the trypan blue exclusion test as described [28, 29]. U937 macrophages were treated with various concentrations of 5, 10, or 50 μM PFOS and PFOA for 24 h or 5 days. Then U937-derived macrophages were washed with PBS, harvested with a cell scraper, and spun down to form a cell pellet. The pellet was then resuspended in 200 µL PBS and 200 µL trypan blue. Cells were incubated for 5 min, and 10 μL of the cell suspension was loaded into a hemocytometer to determine the proportion of nonviable to viable cells. Three individual experiments were performed with four replicate wells per condition.

## Oil Red O (ORO) Staining

If not otherwise indicated, after 5 d of treatment with 10 μM PFOS or PFOA, cells were stained with ORO according to our earlier study [30]. In brief, cells were washed twice with PBS and fixed with 10% formalin for 1 h. After three additional wash steps with water, cells were dried and stained with ORO for 15 min. Excess stain was removed by washing with 70% ethanol; stained cells were then washed with water. Quantification of the stain was performed by dissolving the stained oil droplets in the cell monolayers consisting of 2 × 10^6^ cells with 4% Nonidet P-40 in isopropanol for 5 min. The absorbance was then measured at 520 nm to calculate the amount of accumulated lipids.

## Cellular Cholesterol and Protein Determinations

We extracted free and esterified cholesterol (total cholesterol) directly from macrophage monolayers in situ in the cell culture dish as described earlier [28]. After 5 days of treatment with 5, 10, or 50 μM PFOS and PFOA, cells were washed with PBS and the cell monolayer was scraped off in 400 μL RIPA buffer and incubated for 30 min on ice. Insoluble material was removed by centrifugation at 12,000 × *g* for 20 min at 4 °C and aliquots were used for protein determination according to [31]. The amount of free and esterified cholesterol (total cholesterol) was determined using a colorimetric method (Roche) in the presence of cholesterol oxidase and cholesterol esterase and then measured the absorbance at 405 nm as described [24].

## Cell Migration Assay

We used a six-well Transwell chamber (Costar, Cambridge, MA, USA) to investigate if PFAS exposure would modify cell migration of U937 macrophages. We used a chamber with an upper and lower compartment separated by a 3-µm framed filter. Culture media were filled in the lower compartment, and the upper chamber was prepared with a cell suspension of 1 × 10^6^ macrophages in 1.5 mL medium. Macrophages were preincubated with 10 μM PFOS or PFOA for 24, 48, and 72 h and then added to the upper chamber. The cell migration of macrophages was allowed for 6 h in a 5% CO_2_ tissue culture incubator. After 6 h, the medium of the upper chamber was removed and replaced with 1.5 mL PBS and 20 µM EDTA, and incubated for 30 min at 4 °C. The migrated cells were collected from the lower chamber by centrifugation, and cells that did not migrate were removed from the upper surface of the polycarbonate filter.

## MTT Assay

The reduction of 3-(4,5-dimethylthiazol-2-yl)-2,5-diphenyl tetrazolium bromide (MTT) was measured to quantify the number of cells that migrated into the transwell filter. MTT (5 mg/mL) was added to each well of a six-well plate (100 µL/well in 1.5 mL of complete medium) and incubated at 37 °C for 4 h. After incubation, the medium was removed, and the MTT crystals were solubilized by adding 1 mL of acid-isopropanol. The absorbance of the dissolved MTT crystals was then measured at 540 nm using a spectrophotometer.

## Luciferase Reporter Gene Assay

U937 macrophages were transiently transfected with Nrf2 or PPARγ luciferase reporter plasmids. Nrf2 and PPARγ luciferase reporter plasmids were transfected into U937-derived macrophages as described [32–34]. Briefly, plasmid DNA was transfected into U937 macrophages using Nucleofector technology. 1 × 10⁶ cells were resuspended in 100 μL of Nucleofector Solution V and electroporated with 1.0 μg of plasmid DNA using program V-001 on the Nucleofector device (Amaxa GmbH, Köln, Germany). After 16 h of transfection, the cells were treated for 4 h with 5, 10, or 50 μM PFOS and PFOA as indicated. Luciferase activity was measured using the Dual Luciferase Assay System (Promega, Madison, WI, USA) with a Lumat LB9501 Luminometer (Berthold, Bad Wildbad, Germany). Final data were normalized for transfection efficiency by the relative ratio of firefly and Renilla luciferase activity.

## RNA Isolation and Real-Time PCR

Total RNA was isolated from cells using a Quick-RNA Mini prep isolation kit (Zymo Research, Irvine, CA, USA), and cDNA synthesis was performed as described [28] using a High-Capacity cDNA Reverse Transcriptase Kit (Applied Biosystems, Foster City, CA, USA). RNA was isolated after treatment with 5, 10, or 50 μM PFOS and PFOA in dose-dependent experiments and with 10 μM PFOS and PFOA for 3 to 48 h for time-dependent experiments. Detection of β-actin and differentially expressed target genes was performed with a LightCycler LC480 Instrument (Roche Diagnostics, Indianapolis, IN, USA) using the Fast SYBR Green Master Mix (Applied Biosystems) according to the manufacturer’s instructions. The primers for each gene were designed on the basis of the respective cDNA or mRNA sequences using OLIGO primer analysis software provided by Steve Rozen and the Whitehead Institute/Massachusetts Institute of Technology Center for Genome Research (Table S1). PCR amplification was carried out as described [29]. To confirm the amplification specificity, the PCR products were subjected to melting curve analysis.

## Statistical Analysis

All experiments were performed in triplicate, and results are presented as the mean ± standard deviation (S.D.). Statistical significance was defined as *p* < 0.05. Comparisons between two groups were conducted using an unpaired, two-tailed Student’s *t*-test, while comparisons among multiple groups were evaluated by one-way analysis of variance (ANOVA) followed by either Dunnett’s or Tukey’s post hoc test, as appropriate.

## Results

### Cytotoxicity of PFOS and PFOA

**Fig. 1:**
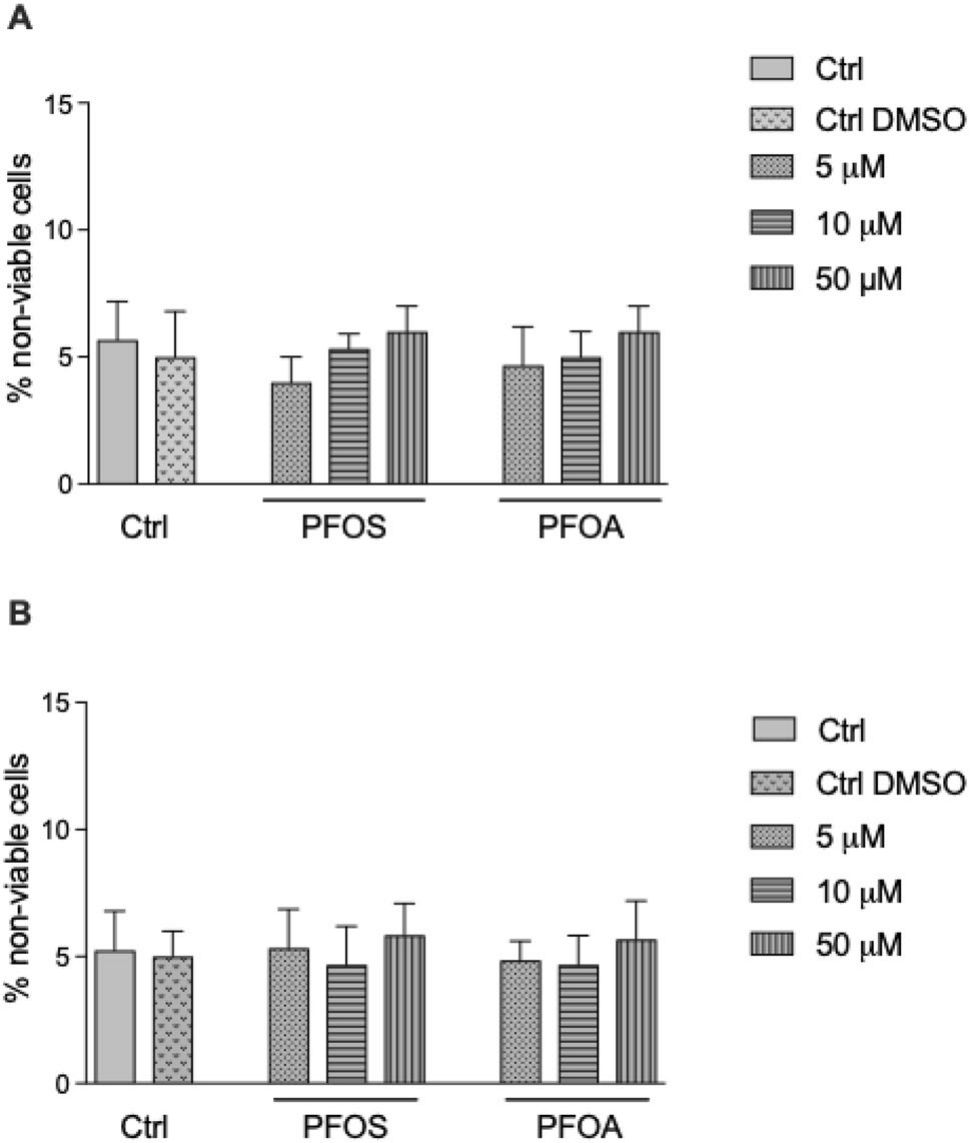
Cell viability of U937-derived macrophages after exposure to PFOS and PFOA. Percent nonviable cells were assessed by trypan blue exclusion test. Macrophages were treated with PFOS or PFOA at 5, 10, and 50 μM concentrations for (**A**) 24 h and (**B**) 5 days. Vehicle control cells received 0.1% DMSO only. Error bars represent mean ± SD of three independent experiments

Cytotoxic effects on U937 macrophages were measured for various concentrations of PFOS and PFOA. The viability of cells cultured in medium alone with 0.1% DMSO added was used as a control. The final concentration of 0.1% DMSO in the vehicle control had no significant effect on cell viability compared to the medium control (Fig. 1). After incubating the cells with PFOS and PFOA for 24 h, the cytotoxicity was measured using the trypan blue exclusion test. The number of dead cells in non-exposed U937 macrophages was about 5%. Cells treated with 5, 10, and 50 μM PFOS or PFOA had no significant effect on cell viability after 24 h of treatment (Fig. 1A). Cells were also treated over a period of 5 days, and cell viability was measured. No significant effect on cell viability was observed by treatment with 5, 10, or 50 μM PFOS or PFOA for 5 days (Fig. 1B). The results confirm studies measuring cytotoxicity in the human monocytic THP-1 cell line observing no cytotoxic effects at concentrations below 100 μM of PFOS or PFOA after 24 h and 48 h of treatment [35].

## Lipid and Cholesterol Accumulation in Macrophages Stimulated by PFOS and PFOA

**Fig. 2:**
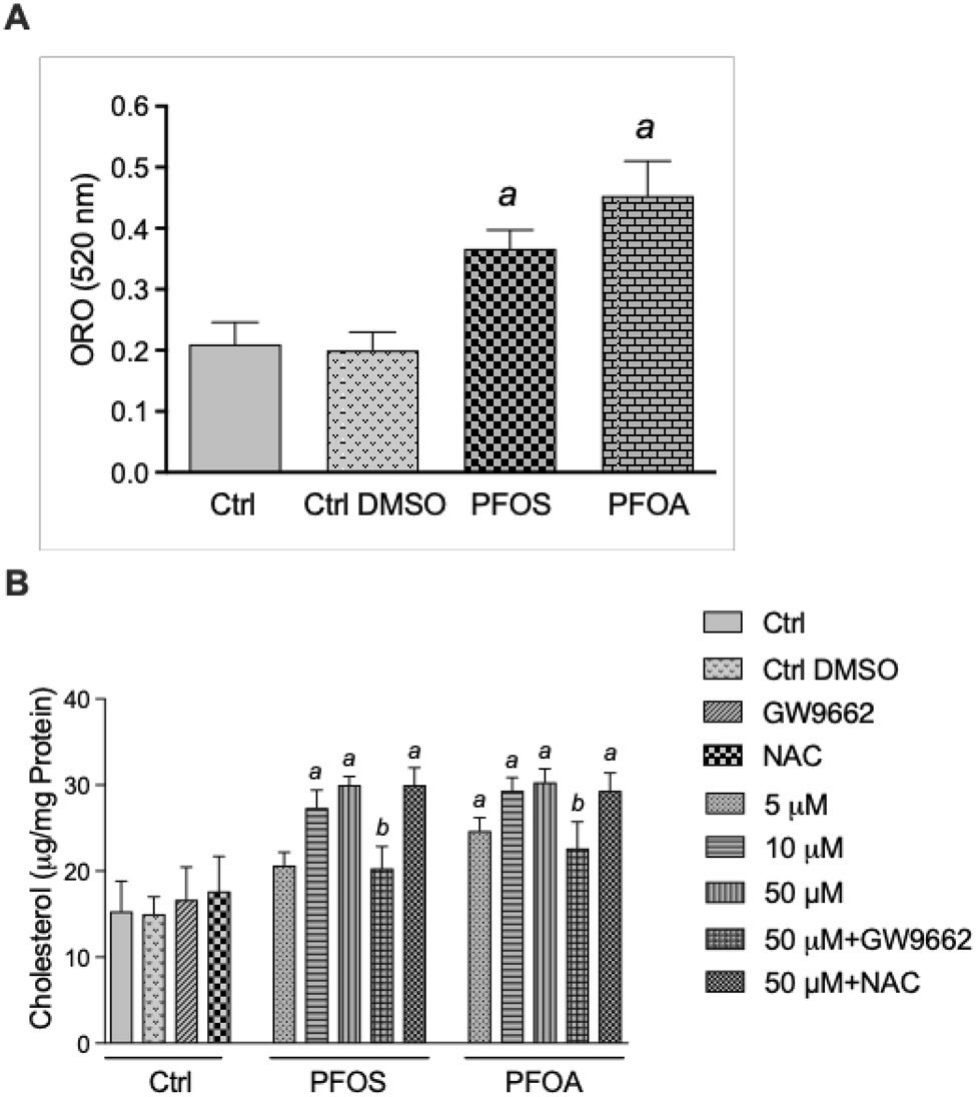
PFAS increases lipid accumulation and cholesterol in macrophages. (**A**) U937-derived human macrophages were treated with PFOS (10 µM) or PFOA (10 µM) for 5 d. Vehicle control cells received 0.1% DMSO. Lipid accumulation was detected by ORO staining at 520 nm and normalized in 2 × 10^6^ cells. (**B**) Cholesterol accumulated in U937 macrophages. Cells were treated for 5 days with 5, 10, or 50 μM PFOS or PFOA. Cells were co-treated with the PPARγ antagonist GW9662 or NAC in the presence of 50 μM PFOS or PFOA. Vehicle control cells received 0.1% DMSO. Total cholesterol was determined using a colorimetric method in the presence of cholesterol oxidase and cholesterol esterase. Values are given as mean ± SD of triplicates of three independent experiments. ^a^Significantly higher than vehicle control (*p* < 0.05). ^b^Significantly lower than cells treated with 50 μM PFOS or 50 μM PFOA (*p* < 0.05)

To quantify lipid accumulation in U937-derived macrophages, we employed the ORO staining method. When macrophages were incubated with 10 µM PFOS or PFOA for 5 days, we observed a 1.9- and 2.3-fold increase in lipid accumulation, respectively, compared to control cells, as measured by spectrophotometric analysis of the eluted ORO staining (Fig. 2A). To quantify the total amount of cholesterol in U937 macrophages, we used a colorimetric method in the presence of cholesterol oxidase and cholesterol esterase. Macrophages were exposed to 5, 10, and 50 μM PFOS or PFOA for 5 days. Treatment with 10 μM and 50 μM PFOS stimulated the accumulation of cholesterol by 1.8- and 2.0-fold, respectively (Fig. 2B). PFOA concentration-dependently increased the amount of cholesterol with a maximal increase at 10 and 50 μM of 2.0-fold above control. Results from the cholesterol assay were consistent with findings from ORO (Fig. 2A). Co-treatment with the PPARγ antagonist GW9662 effectively reduced the cholesterol accumulation in macrophages treated with 50 µM PFOS or PFOA. Treatment with GW9662 only had no effect on the level of cholesterol. Additionally, macrophages were treated with N-Acetylcysteine (NAC), used to neutralize oxidative stress. NAC did not affect the accumulation of cholesterol induced by PFOS or PFOA. Treatment with NAC only had no effect on cholesterol accumulation compared to the vehicle control. The treatment with 0.1% DMSO in the vehicle control did not significantly change the accumulation of lipids or cholesterol compared with a medium control (Fig. 2A, B).

## Activation of PPARγ and Nrf2 Signaling by PFOS and PFOA

**Fig. 3:**
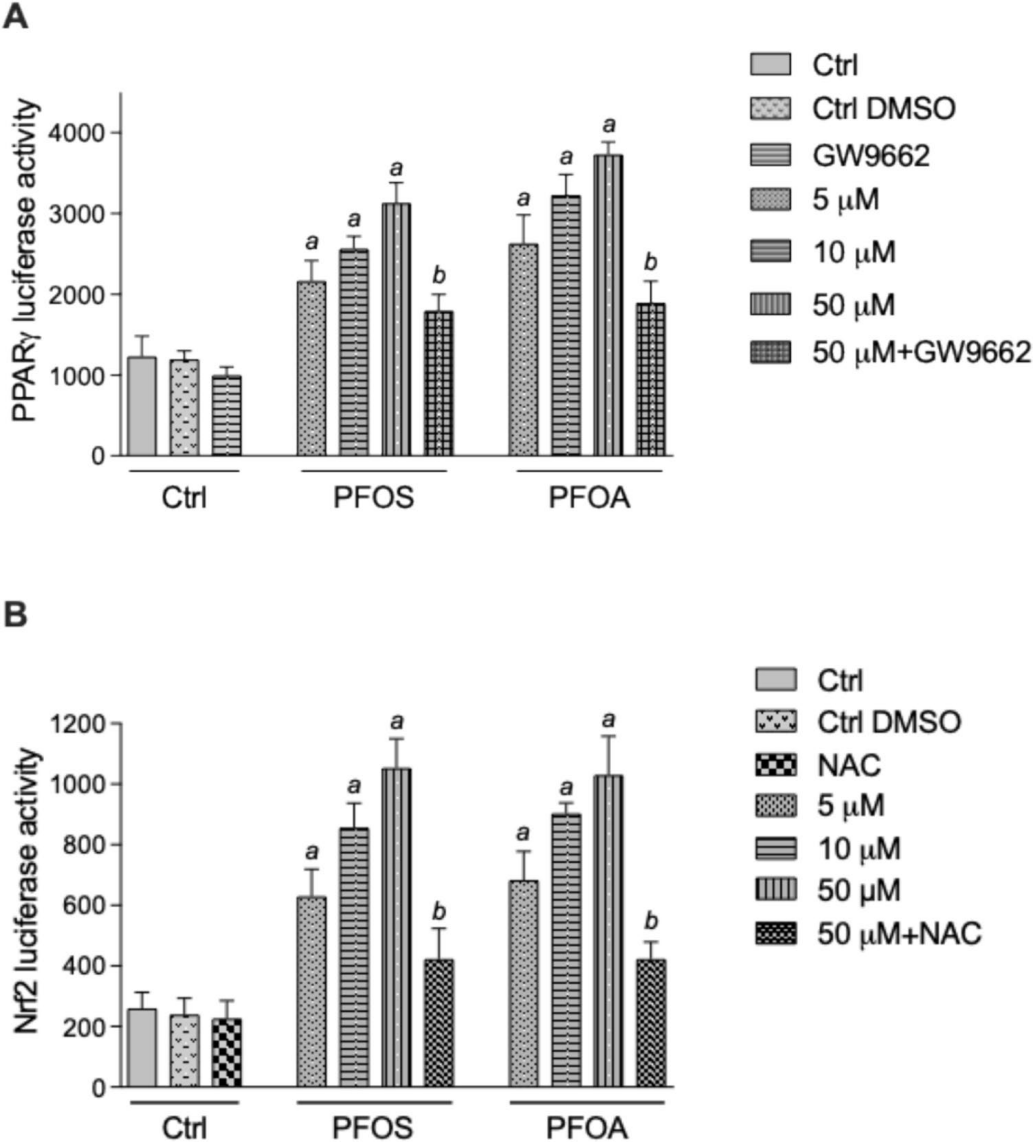
PFOS and PFOA increase PPARγ and Nrf2 activity. U937-derived macrophages were transiently transfected with a (**A**) PPARγ or (**B**) Nrf2 luciferase reporter plasmid. After 16 h the cells were treated for 4 h with various concentrations of PFOS or PFOA (5, 10, and 50 μM). Additionally, PPARγ-transfected cells were treated with the PPARγ antagonist GW9662 (30 nM) in the presence of 50 μM PFOS or 50 μM PFOA. Nrf2-transfected cells were co-treated with the ROS scavenger NAC (1 mM) in the presence of 50 μM PFOS or 50 μM PFOA.^a^Significantly higher than control cells (*p* < 0.05). ^b^Significantly lower than PFOS- and PFOA-treated cells (*p* < 0.05)

Studies have shown that PFAS chemicals activate the PPAR signaling pathways [36]. PPARs regulate lipid metabolism and play a key role in the development and progression of atherosclerosis. The luciferase assay results show that PPARγ transcriptional activity was increased in a concentration-dependent manner by PFOS and PFOA (Fig. 3A). PFOS and PFOA induced the PPARγ activity at 5 μM and 10 μM concentrations, which was further increased at 50 μM after treatment for 4 h (Fig. 3A). The PPARγ antagonist, GW6992, effectively reduced the activity of PPARγ induced by PFOS and PFOA. Additionally, PFOS and PFOA concentration-dependently activated the nuclear factor Nrf2, which regulates the transcriptional activity of the oxidative stress responsive enzymes such as heme oxygenase 1 (HMOX1) and the antioxidant enzyme NAD(P)H:quinone oxidoreductase (NQO1) (Fig. 3B). NAC, a scavenger of reactive oxygen species (ROS), inhibited the PFOS- and PFOA-induced activation of Nrf2. The vehicle control with DMSO at 0.1% did not significantly change the basal level of PPARγ or Nrf2 activity.

## Modified Expression of Atherogenic and Inflammatory Marker Genes Induced by PFOS and PFOA

**Fig. 4:**
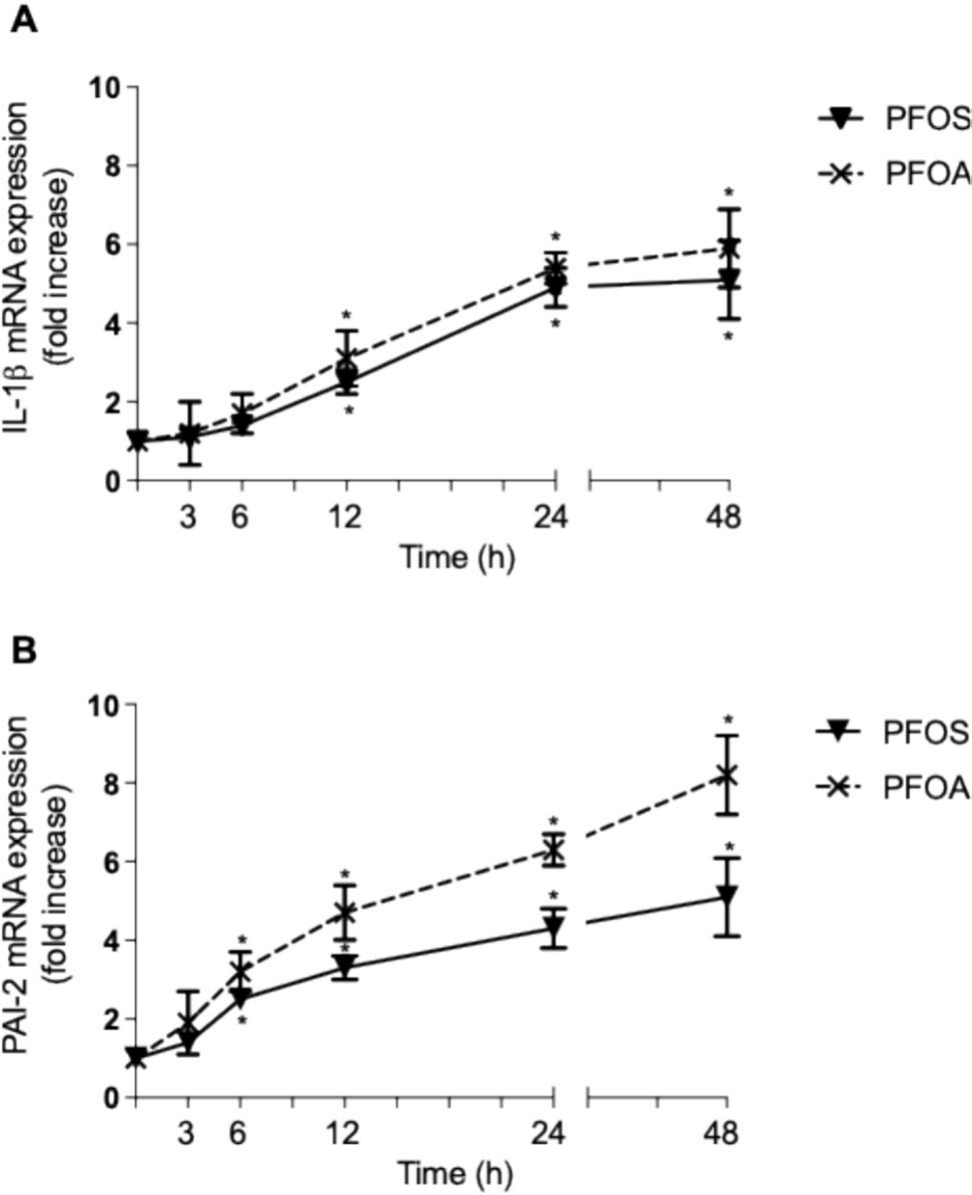
Time-course study of (**A**) IL-1β and (**B**) PAI-2 mRNA expression in U937-derived human macrophages. Macrophages were treated for 3, 6, 12, 24, and 48 h with 10 μM PFOS or 10 μM PFOA. Control cells of each time point received 0.1% DMSO. mRNA expression is shown as fold increase compared to the respective controls. *Significantly higher than control (*p* < 0.05)

**Fig. 5:**
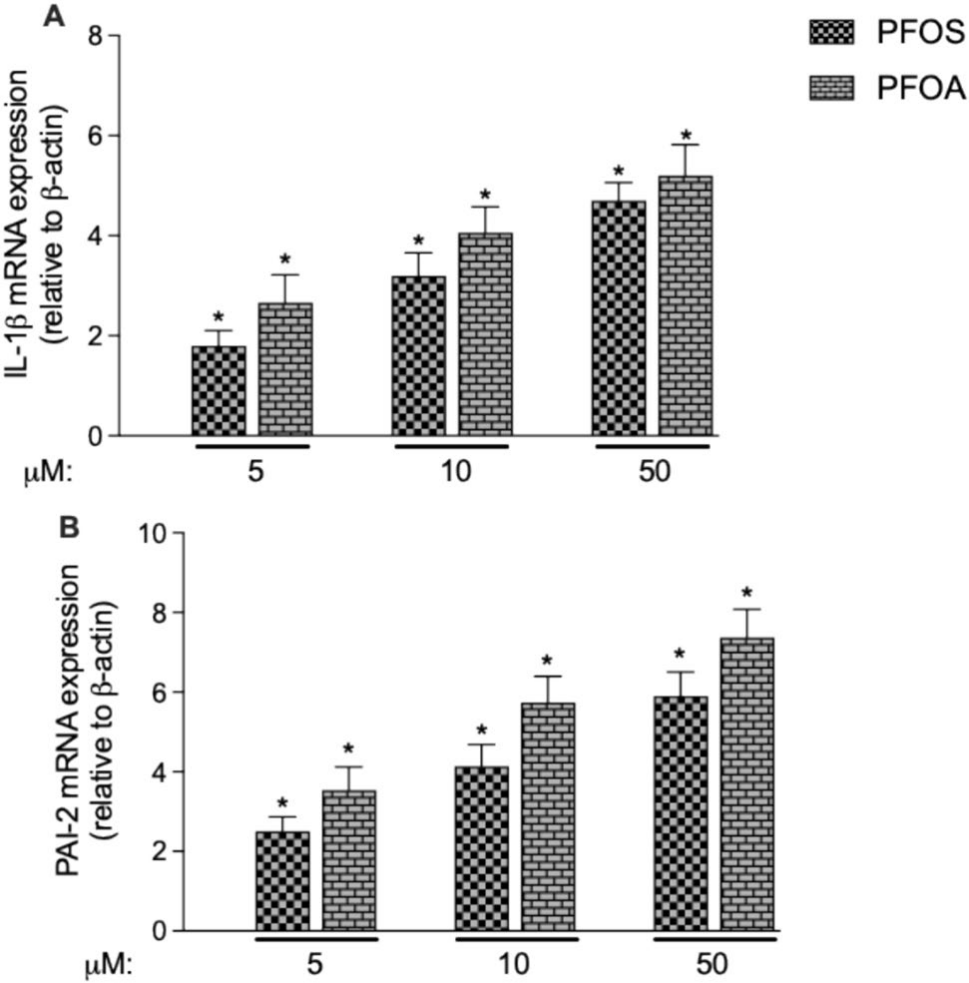
Concentration-dependent induction of (**A**) IL-1β and (**B**) PAI-2 mRNA. U937 macrophages were treated with 5, 10, and 50 μM PFOS or PFOA for 24 h, and mRNA was analyzed by real-time RT-PCR. Control cells received 0.1% DMSO used as vehicle. Results are normalized to β-actin and given as relative changes of the mRNA levels in PFAS-treated cells vs. control. *Significantly different from control (*p* < 0.05)

*Time- and concentration-dependent increase of IL-1β and PAI-2*. Next, we tested the effect of PFOS and PFOA on the expression of genes which are critically involved in activation of macrophages and the process of atherosclerosis. First, we analyzed the expression of IL-1β and PAI-2 in PFAS-treated U937-derived macrophages. IL-1β plays a central role in inflammatory processes and has been associated with atherosclerotic plaque formation [37, 38]. PAI-2 plays a role in atherosclerosis and foam cell formation through its effects on inflammation and extracellular matrix remodeling [39]. PAI-2 is expressed in inflammatory macrophages. The overexpression of PAI-2 can lead to increased macrophage activity and enhanced foam cell formation, further accelerating the development of atherosclerotic plaques [40]. To assess the time course of PFAS-induced changes of IL-1β and PAI-2 expression, we treated U937 macrophages with 10 µM PFOS or PFOA and analyzed the mRNA expression at various time points (3, 6, 12, 24, and 48 h). The results (Fig. 4) show that both PFOS and PFOA significantly increased the expression of IL-1β and PAI-2. The earliest time point of a significant increase (about twofold) of IL-1β occurred after 12 h of treatment with PFOS or PFOA. The expression of IL-1β was increased fourfold above control at 24 and 48 h of PFOS treatment. PFOA led only to slightly higher increase of PAI-2 at 48 h compared to PFOS. PFOS and PFOA induced PAI-2 expression 2.2-fold and 3.0-fold at 6 h, respectively. The expression of PAI-2 further increased at 12 and 24 h treatment with 10 µM PFOS or PFOA. At the latest time point tested, PFOS and PFOA increased the level of PAI-2 mRNA fivefold and eightfold, respectively. Following the time-dependent increase, we tested various concentrations (5 to 50 µM) of PFOS and PFOA. After 24 h of treatment, expression of IL-1β was concentration-dependently upregulated at 5, 10, and 50 µM PFOS and PFOA (Fig. 5A). IL-1β mRNA level increased about 4.3-fold above control at 50 µM PFOS and PFOA. No significant increase of IL-1β was observed at the lowest concentration of 1 µM PFOS or PFOA (data not shown). The expression of PAI-2 was upregulated by PFOS and PFOA after 24 h treatment. PFOS induced PAI-2 expression by 2.5-, 4.0-, and 6.0-fold and PFOA led to a 3.8-, 6.0-, and 7.8-fold increase of PAI-2 at 5,10, and 50 µM (Fig. 5B).

*Effect of PFOS and PFOA on expression of genes involved in cholesterol metabolism and atherogenesis.* Furthermore, macrophages were treated with PFOS or PFOA to analyze selected markers which are critically involved in cholesterol metabolism and atherogenesis. Cells were treated for 48 h with 10 µM PFOS or PFOA. Here, we found a 2.0-fold increase of the ATP-binding cassette transporter A1 (ABCA1) after PFOS and a 3.8-fold increased level after treatment with PFOA (Table 1). ABCA1 is known to regulate cholesterol efflux, and an inappropriate expression of ABCA1 in macrophages can lead to cholesterol accumulation and foam cell formation [41]. We also found an increased expression of AKR1C3 of 2.9-fold by PFOS and a 3.8-fold increase by PFOA after 48 h treatment. AKR1C3 has been found to be involved in the development of hormone-dependent cancers including breast, prostate, and ovarian cancers. Moreover, the enzyme activity of AKR1C3 has been shown to modulate inflammatory responses in macrophages and enhance the uptake and accumulation of cholesterol within macrophages induced by oxidized low-density lipoprotein [42]. Furthermore, treatment with PFOS or PFOA induced a respective 2.7- and 3.2-fold increase in COX-2 mRNA expression relative to the control treatment. COX-2 has been found to be an important enzyme regulating cholesterol metabolism and foam cell formation in macrophages [43]. Upregulation of COX-2 may enhance or regulate lipid accumulation and foam cell formation [44, 45]. Interestingly, the expression of IL-6, an indicator of acute inflammation, was significantly suppressed by PFOS and PFOA (Table 1). IL-6 has been found to be involved in endothelial dysfunction and plaque formation [46]. Additionally, we analyzed the expression of enzymes involved in cholesterol metabolism such as CYP8B1 [47, 48] and LSS [49]. LSS is a key enzyme in the cholesterol synthesis pathway and CYP8B1 in bile acid synthesis from cholesterol. Both enzymes have been recently described to be concentration-dependently suppressed after exposure to PFOA and PFOS in the HepaRG cell line [50]. In U937-derived macrophages, a 60% decrease in CYP8B1 expression was observed following a 48 h exposure to PFOS or PFOA. Similarly, we found a 40% reduction in LSS expression in PFOS-treated cells, and a 50% reduction in LSS expression in PFOA-treated cells. Macrophage treatment with PFOS and PFOA showed a significant increase of heme oxygenase 1 (HMOX1) and NAD(P)H:quinone oxidoreductase (NQO1). Furthermore, we analyzed the expression of MMPs, which have been identified to contribute to various health issues such as chronic inflammation and atherosclerosis [51]. Increased levels of MMPs have been described during foam cell formation in our earlier studies [30, 52]. PFOS and PFOA induced the expression of MMP-1 by 2.3- and 4.2-fold, respectively, in U937-derived macrophages. We also observed a significant upregulation of MMP-12 by PFOS and PFOA. The vehicle control received 0.1% DMSO which did not significantly change the expression of the genes tested compared to the medium control (Table S2). Furthermore, we co-treated cells with the PPARγ antagonist GW9662 or NAC to test if PPARγ activation or generation of oxidative stress would have an impact on PFOS- or PFOA-induced changes in gene expression (Table 1). The changes in mRNA levels induced by PFOS and PFOA on ABCA1 and IL-6 were reversed when cells were co-treated with GW9662. The elevated levels of HMOX1 and NQO1 mRNA induced by PFOS and PFOA were significantly suppressed in the presence of NAC. The single treatment with GW9662 or NAC had no significant effect on the basal expression level of the target genes tested compared to untreated control cells (Table. S2).

**Table 1:** Expression of key enzymes and genes involved in inflammation, cholesterol synthesis, and oxidative stress

*Gene*	PFOS	PFOA	PFOS	PFOA	PFOS	PFOA
	-	-	+ GW9662	+ GW9662	+ NAC	+ NAC
*ABCA1*	2.0^a^	3.8^a^	1.3^b^	2.5^b^	1.8^a^	3.4^a^
*AKR1C3*	2.9^a^	3.8^a^	2.6^a^	3.5^a^	2.8^a^	3.7^a^
*CYP8B1*	0.4^a^	0.4^a^	0.5^a^	0.3^a^	0.3^a^	0.4^a^
*COX-2*	2.7^a^	3.2^a^	2.4^a^	3.0^a^	2.5^a^	2.8^a^
*HMOX1*	3.5^a^	4.5^a^	3.3^a^	4.1^a^	1.3^b^	1.4^b^
*IL-6*	0.3^a^	0.2^a^	1.1^b^	0.9^b^	0.2^a^	0.3^a^
*LSS*	0.6^a^	0.5^a^	0.4^a^	0.4^a^	0.3^a^	0.5^a^
*MMP-1*	2.3^a^	4.2^a^	2.4^a^	3.6^a^	2.1^a^	3.9^a^
*MMP-12*	3.3^a^	4.5^a^	3.0^a^	4.1^a^	3.4^a^	4.4^a^
*NQO1*	2.8^a^	3.0^a^	2.5^a^	2.8^a^	1.1^b^	1.3^b^

U937-derived human macrophages were treated for 48 h with 10 μM PFOS or PFOA in the presence or absence of GW9662 or NAC. mRNA expression was analyzed by real-time PCR and is normalized to the expression of β-actin. Results of three separate experiments are shown as means of fold change compared to vehicle control. ^a^Significantly different from control (*p* < 0.05); ^b^Significantly different from PFOS- or PFOA-treated cells (*p* < 0.05)

## PFOS and PFOA Induce Cell Migration of U937-Derived Macrophages

**Fig. 6:**
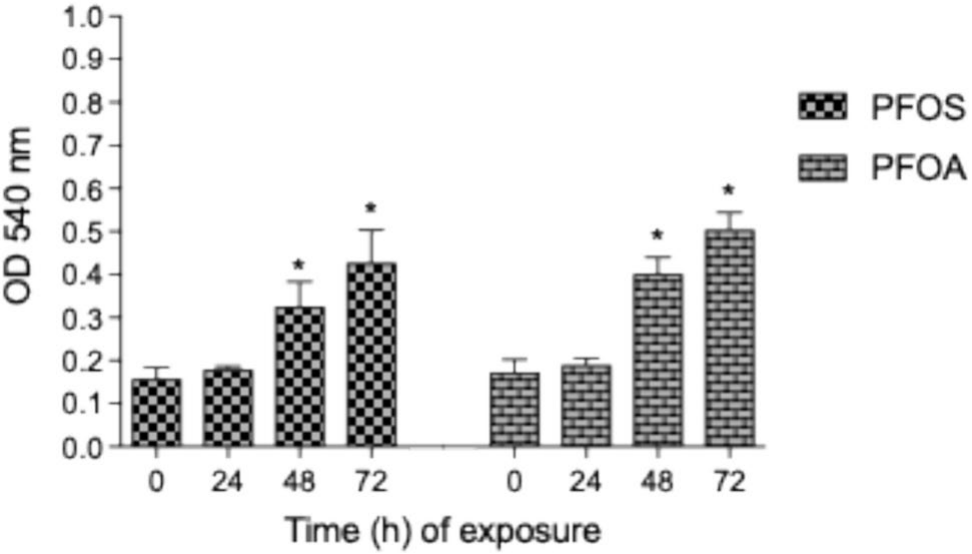
PFOS and PFOA exposure stimulates chemotaxis of U937-derived macrophages. Cells were treated with 10 µM PFOS or 10 µM PFOA for 24, 48, or 72 h, after which they were placed in the upper wells of a chemotaxis chamber. The cells were allowed to migrate for 6 h, followed by an MTT assay. The plate was then read at 540 nm to determine the number of migrated cells. Results of three separate experiments are presented as means ± SD. *Indicates a significant increase compared to the 0-h time point (*p* < 0.05)

Increased chemotaxis and migration are linked to pathological conditions, such as inflammation, tumor metastasis, and cardiovascular diseases. Therefore, we tested the effect of PFOS and PFOA on cell migration of U937-derived macrophages. Treatment of U937 macrophages with 10 µM PFOS or PFOA for 24 h did not affect cell migration. However, cell motility was significantly enhanced after 48 h and 72 h of treatment with PFOS or PFOA (Fig. 6).

At 48 h, cells treated with either PFOS or PFOA both experienced a 45% increase in migration relative to the vehicle control. At 72 h, this further increased to 70% (Fig. 6). There was no significant difference in cell motility between PFOS- or PFOA-treated cells in a Transwell chamber.

## Effect of PFOS and PFOA on IL-1β, IL-6, and COX-2 in SARS-CoV-2 Spike S1 Activated Macrophages

**Fig. 7:**
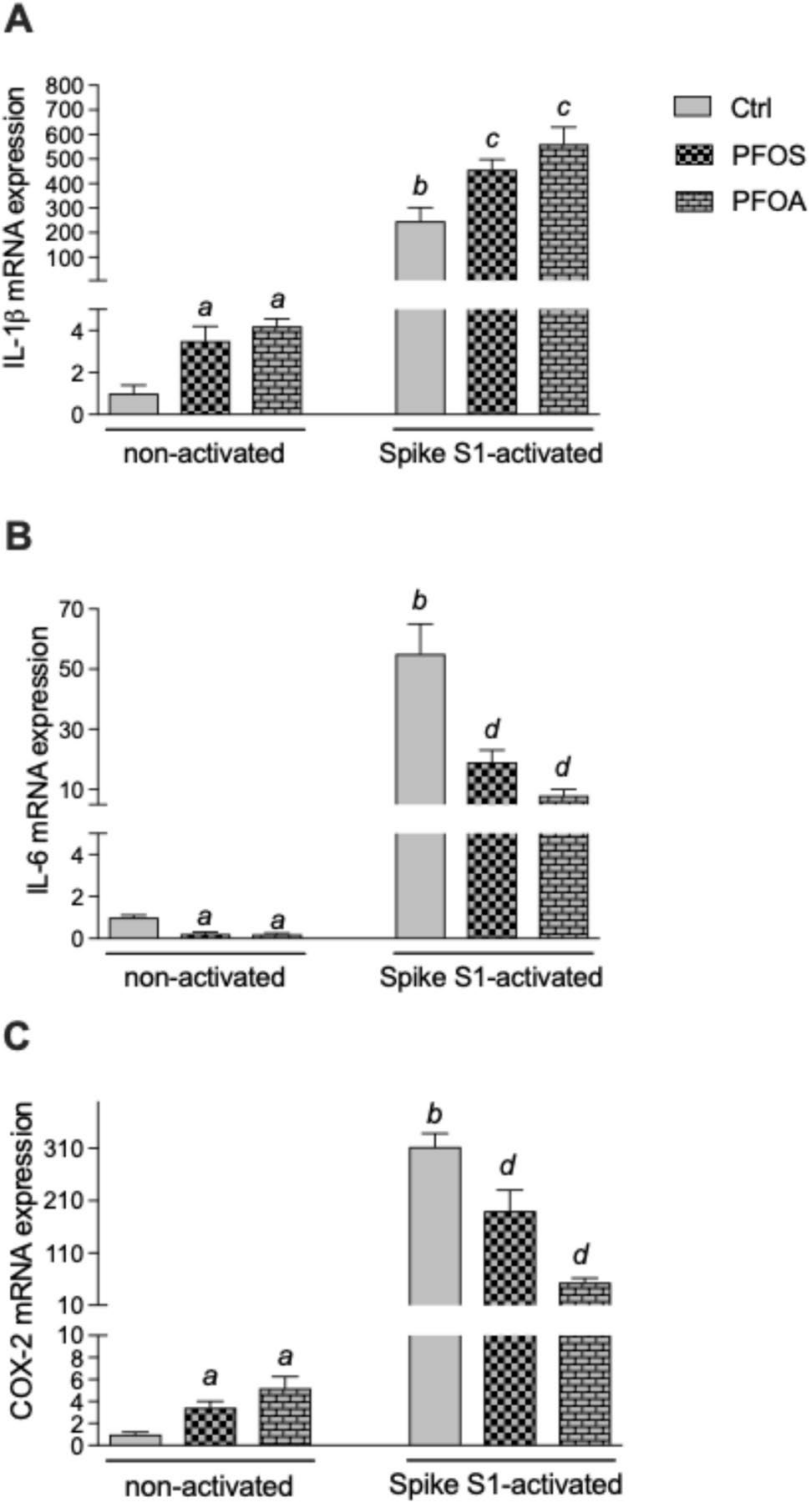
Modulation of inflammatory markers in activated U937-derived macrophages by PFOS and PFOA. Cells were treated with 10 μM PFOS or 10 μM PFOA for 24 h in the absence or presence of spike S1 protein of SARS-CoV-2. (**A**) IL-1β mRNA, (**B**) IL-6 mRNA and **(C)** COX-2 mRNA were analyzed by real-time RT-PCR and normalized to the expression of β-actin. Results of three separate experiments are presented as means ± SD. ^a^Significantly different from non-activated control (*p* < 0.05). ^b^Significantly higher than non-activated control (*p* < 0.05). ^c^Significantly higher than cells treated with spike S1 only, (*p* < 0.05).^d^Significantly lower than cells treated with spike S1 only, (*p* < 0.05)

The results of the expression analysis showed that PFOS and PFOA suppress the expression of IL-6, whereas the expression of IL-1β and COX-2 is increased after treatment with PFOS and PFOA in steady-state macrophages. To study the impact of PFAS on activated macrophages, we tested the effect of PFOS and PFOA when U937-derived macrophages become activated with SARS-CoV-2 spike S1 protein. The results show that the SARS-CoV-2 Spike S1 significantly activated the expression of IL-1β, IL-6, and COX-2 (Fig. 7). Treatment with PFOS and PFOA significantly enhanced the expression of IL-1β in spike S1-activated macrophages (Fig. 7A). However, the expression of IL-6 and COX-2 was repressed when spike S1-activated cells were co-treated with PFOS or PFOA (Fig. 7B, C). PFOS and PFOA inhibited the expression of spike S1-induced IL-6 by about 70% and 85%, respectively. The spike S1-induced expression of COX-2 was reduced by 35% and 80% after treatment with PFOS and PFOA.

## Discussion

PFAS are now found globally in water, soil, and human tissues. Growing evidence from epidemiological and experimental studies suggests that PFAS exposure is associated with adverse metabolic effects, particularly disruptions in lipid metabolism [13, 53]. The results of the current study show that exposure to PFOS and PFOA stimulates the accumulation of cholesterol in U937-derived macrophages. Recent reports, including our own studies, have shown that U937-derived macrophages respond to oxidized low-density lipoprotein and cholesterol mediating the formation of cholesterol-rich foam cells associated with the release of pro-inflammatory cytokines [28, 30, 54, 55]. Furthermore, we found similar responses to inflammatory stimuli in primary human monocyte-derived dendritic cells compared to U937-derived dendritic cells [56] suggesting that the human U937-derived macrophages serve as a suitable model to study the response to lipids and inflammatory stimuli.

Accumulation of cholesterol in macrophages is a pathway to foam cell formation, a hallmark in atherogenesis [57]. The results confirm findings in humans showing elevated PFAS serum levels linked with increased total cholesterol and low-density lipoprotein (LDL) cholesterol, both of which are established risk factors for CVD [23]. A strong association of PFAS exposure with high levels of cholesterol has been observed also in mechanistic and in vivo studies as well as epidemiological reports [15–21]. These associations raise significant public health concerns, as chronic PFAS exposure may contribute to the development of atherosclerosis, hypertension, and other cardiovascular conditions, underscoring the need for further research and regulatory action. The underlying mechanisms leading to alterations in blood lipids through PFAS exposure in humans are not well understood. PFAS chemicals are known to interact with protein members of the PPAR family including PPARα and PPARγ. PFOA and PFOS have been found to activate PPARα and PPARγ [36, 58–63]. PPAR signaling pathways are critical players in fatty acid metabolism [61, 64]. We found an increase of PPARγ reporter activity after treatment of U937-derived macrophages with PFOS and PFOA. The treatment of U937 macrophages with a PPARγ antagonist suppressed the PFAS-stimulated accumulation of cholesterol by about 60%. The results suggest that the PFAS-induced cholesterol accumulation in macrophages is partly mediated by PPARγ. PPARγ has been shown to contribute and alter lipid metabolism, which is associated with lipid accumulation in macrophages [65].

Additionally, we analyzed the activation of Nrf2 by PFAS. Previous studies have demonstrated that exposure to PFAS mediates the generation of oxidative stress in various cell types [66, 67]. Oxidative stress activates the transcription factor Nrf2, a key regulator of markers of oxidative stress and antioxidant enzymes [68]. Our results show that PFOS and PFOA activate Nrf2 signaling, which is repressed when cells were co-treated with NAC, a scavenger of ROS. The results indicate that PFOS and PFOA induce oxidative stress in macrophages, confirming previous studies in HepG2 and endothelial cells [66, 67]. Our results are supported by the expression analysis showing increased mRNA levels of Nrf2-regulated genes such as HMOX1 and NQO1 in PFOS- and PFOA-treated U937-derived macrophages. Moreover, the generation of oxidative stress may lead to lipid peroxidation and activation of macrophages, consequently increasing pro-inflammatory and proatherogenic effects and contributing to the progression of atherosclerosis and other inflammatory diseases [69, 70].

Macrophages and foam cells express inflammatory cytokines as well as metabolic enzymes [71]. Cytokines can contribute to the initiation and progression of atherosclerotic lesions by triggering multiple cellular functions such as leukocyte recruitment and the synthesis or degradation of extracellular matrix [72]. Here, we found that PFOS and PFOA induced mRNA expression of IL-1β in U937-derived macrophages in a time- and concentration-dependent manner. The concentrations of PFOS and PFOA measured in human serum concentrations are usually between 1 and 100 ng/mL, translating to about 2–200 nM. Thus, the concentrations of PFOS and PFOA used in the current study are higher than what is usually found in human blood. However, in vitro studies with human epithelial breast cells showed significant effects at low concentrations of 100 pM PFOS or PFOA on mechanisms implicated in tumorigenesis, including cell migration and invasion [73]. Additionally, the bioaccumulation of PFAS in certain tissues and cell types may significantly increase the concentrations found in human blood. Our current data confirm previous studies showing increased expression and protein production of IL-1β by PFAS in vitro as well as in vivo [74–76]. IL-1β is involved in various stages of atherosclerotic plaque formation, including endothelial dysfunction, immune cell recruitment, and lipid metabolism [37]. IL-1β can directly activate macrophages and enhance their capacity to accumulate lipids and the formation of foam cells [77]. Furthermore, IL-1β contributes to the destabilization of atherosclerotic plaques by inducing the expression of MMPs [78]. In contrast to IL-1β, we found a downregulation of IL-6, a marker which may contribute to plaque formation [46]. Interestingly, the downregulation of IL-6 by PFOS and PFOA was abrogated in the presence of a PPARγ antagonist. Our data confirm a previous study showing the downregulation of IL-6 by PPARγ ligands in pancreatic acinar cells [79]. Additionally, PFOS and PFOA exposure time- and concentration-dependently induced the expression of PAI-2 in U937-derived macrophages. Both PAI-1 and PAI-2 are serine protease inhibitors and inhibitors of plasminogen activators, such as tissue-type plasminogen activator (tPA) and urokinase (uPA) [80, 81]. By inhibiting plasminogen activators, the PFAS-induced expression of PAI-2 can result in foam cell formation and the formation of plaques, increasing the risk of cardiovascular events as described for PAI-1 [40, 82].

Furthermore, we observed increased levels of ABCA1 and AKR1C3 in PFOS- and PFOA-treated macrophages. The PFAS-induced increase of ABAC1 was abolished when PPARγ was antagonized. A previous study showed that activation of PPARγ induces LXRα expression, which in turn upregulates ABCA1 in macrophages [83]. ABCA1 and AKR1C3 can regulate inflammatory pathways within macrophages, leading to an enhanced uptake of modified LDL and accumulation of cholesterol [41, 42]. AKR1C3 has also been shown to convert androgens to estrogens, influencing hormone signaling and promoting the growth of hormone-dependent cancers, including breast, prostate, and ovarian cancers [84–86]. AKR1C3 has been found to be overexpressed in estrogen receptor-positive (ER +) breast cancers [87]. Interestingly, the risk to develop hormone receptor-positive breast cancer has been associated with elevated PFAS serum levels [88–90]. On the other hand, we found suppressed expression of CYP8B1 and LSS after treatment with PFOS and PFOA, confirming a recent study with hepatocytes derived from a hepatocellular carcinoma [50]. CYP8B1 and LSS are both involved in cholesterol metabolism, and PFAS-mediated reduction of the activity of these enzymes may contribute to cholesterol accumulation in macrophages [48, 49]. Further, we found an upregulation of COX-2 by PFOS and PFOA in macrophages, which may enhance the degradation of the extracellular matrix and may contribute to plaque instability. A previous study demonstrated that the contribution of COX-2 expression by activated macrophages was associated with increased activity of MMPs and acute ischemic syndromes and plaque rupture [91]. Here we found an increased expression of MMP-1 and MMP-12 associated with stimulated cell migration of macrophages induced by PFOS and PFOA, which supports the role of macrophages in PFAS-mediated development of foam cells and atherosclerosis. MMPs can degrade the extracellular matrix (ECM) and weaken the structural integrity of the plaques [92, 93]. This can lead to plaque rupture, which is a critical event in the development of acute cardiovascular events, such as heart attacks and strokes.

To further analyze the effect of PFAS on inflammatory responses, we activated U937-derived macrophages with recombinant spike S1 protein of SARS-CoV-2. The spike S1 protein was simply used as a tool to investigate the effects of PFAS on activated macrophages and to examine the impact of PFAS under inflammatory conditions. Infection with SARS-CoV-2 has been linked to various atherosclerotic cardiovascular complications associated with dysregulated blood lipid levels and chronic inflammation [94–99]. Our results show that activation of macrophages with spike S1 significantly induced the pro-inflammatory markers IL-1β, IL-6, and COX-2. However, the elevated levels of IL-6 and COX-2 induced by spike S1 of SARS-CoV-2 were significantly repressed when macrophages were co-treated with PFOS or PFOA. On the other hand, exposure to PFOS and PFOA significantly enhanced the expression of IL-1β in activated U937-derived macrophages. As shown, PFOS and PFOA increased the expression of COX-2 in non-activated macrophages; however, PFOS and PFOA surprisingly suppressed COX-2 in spike S1-activated macrophages. The underlying mechanism of the divergent effect is not clear. It is possible that PFAS-induced signaling pathways interfere with key signaling pathways involved in inflammation and induced by pathogens. Depending on the gene’s specific regulatory mechanisms, its expression may be either upregulated or downregulated by PFAS when cells are activated by inflammatory stimuli. Consequently, our data clearly indicate the dysregulation of inflammatory responses induced by PFAS exposure in macrophages. The results confirm previous studies showing that the spike protein of the SARS coronavirus significantly modulates inflammatory responses in macrophages which are associated with SARS-CoV-2 infection and cardiovascular events [100–104]. Furthermore, PFAS chemicals are known to modulate immune responses and show many characteristics of chemicals inducing immunotoxicity [105–110]. Moreover, several studies found a positive link between elevated PFAS blood levels and COVID-19 incidences and the severity of symptoms [111–115]. These studies indicate how exposure to PFAS may further contribute to the development of atherosclerotic cardiovascular diseases.

In conclusion, PFOS and PFOA induced lipid accumulation associated with increased PPARγ and Nrf2 activity, including changes in the expression of enzymes and markers of inflammation and oxidative stress, which are critically involved in the development of atherosclerosis and CVD. PFOS and PFOA also modified inflammatory responses in macrophages activated by spike S1 of SARS-CoV-2, suggesting their potential as immunomodulatory agents that impair important immune functions, further contributing to the development of atherosclerotic cardiovascular diseases.

## Supplementary Information

Below is the link to the electronic supplementary material.

Supplementary file1 (PDF 174 kb)

## Data Availability

No datasets were generated or analyzed during the current study.
