# Supplementary material for: Per- and Polyfluoroalkyl Substances (PFAS) Enhance Cholesterol Accumulation and Dysregulate Inflammatory Responses in Macrophages
Source: Cardiovasc Toxicol. 2025 Jul 29;25(10):1455–70. doi: 10.1007/s12012-025-10048-w (PMC12432094; doi:10.1007/s12012-025-10048-w)
Supplement: Supplementary file 1 — Supplementary file1 (PDF 174 kb) [file 12012_2025_10048_MOESM1_ESM.pdf]

Supplementary Information

Per- and polyfluoroalkyl substances (PFAS) enhance cholesterol accumulation and dysregulate inflammatory responses in macrophages

Journal: Cardiovascular Toxicology

Jack C. Connolly[1], Yasuhiro Ishihara[2], Emma Sawaya[1], Valerie Whitfield[1], Nicole Garrity[1], Rajveer Sohata[1], Mark Tsymbal[1], Alyssa Lundberg[1], Michele A. La Merrill[3], Jamie C. DeWitt[4], Allison K. Ehrlich[3], and Christoph F. A. Vogel[1,3,#]

1. Center for Health and the Environment, University of California, Davis, CA
2. Program of Biomedical Science, Graduate School of Integrated Sciences for Life, Hiroshima University, Hiroshima, 739-8521, Japan.
3. Department of Environmental Toxicology, University of California, Davis, CA
4. Department of Environmental and Molecular Toxicology, Oregon State University, Corvallis 97331, Oregon, United States.

#Correspondence: Christoph F. A. Vogel, cfvogel@ucdavis.edu, ORCID# 0000-0002-7561-4598

Table S1

Table S1. PCR primers for human target genes

Gene	**Forward Primer**	**Reverse Primer**	**Product (bp)**
β-actin	catccgcaaagacctgtacg	cctgcttgctgatccacatc	218
ABAC1	tgagaggaagttctgggctg	cgtaccgcatgtcctcaaag	182
AKR1C3	atttggcacctatgcacctc	cacactgccatctgcaatct	163
COX-2	ggaacacaacagagtatgcg	aaggggatgccagtgataga	195
CYP8B1	accctgaagatgtccagtgg	tggtggatcttcttgcctgt	191
HMOX1	attctcttggctggcttcct	cccctctgaagtttaggcca	153
IL-1β	gggcctcaaggaaaagaatc	ttctgcttgagaggtgctga	205
IL-6	agtcctgatccagttcctgc	ctacatttgccgaagagccc	196
LSS	gtcttctgtccagctccctt	cccaacacagttccttcagc	165
MMP1	caagcattgggtgtttgatg	tgaatgaccctctgggagac	248
MMP12	acacatttcgcctctctgct	ccttcagccagaagaacctg	192
NQO1	tcccaggttccagcaattct	cactttgggaggctgaggta	177
PAI-2	ggttcatgcagcagatccag	aagtctactgcctggggttc	235

Table S2

Table S2. Expression of key enzymes and genes involved in inflammation, cholesterol synthesis, and oxidative stress.

Gene	DMSO	GW9662	NAC
ABAC1	0.9	0.8	1.2
AKR1C3	1.0	1.1	1.4
CYP8B1	1.2	1.0	1.3
COX-2	0.9	1.2	1.0
HMOX1	0.8	1.3	0.8
IL-6	1.3	1.2	0.9
LSS	0.7	0.9	1.4
MMP-1	1.2	1.1	1.2
MMP-12	1.3	1.0	1.1
NQO1	0.8	1.2	0.9

U937-derived human macrophages were treated for 48h with DMSO (0.1%), GW9662 (30 nM), or NAC (1 mM). mRNA expression was analyzed by real-time PCR and is normalized to the expression of β-actin. Results of three separate experiments are shown as fold change compared to a medium control and as means ± SD.
